# Missed opportunities for HIV testing in people diagnosed with HIV, Estonia, 2014 to 2015

**DOI:** 10.2807/1560-7917.ES.2019.24.15.1800382

**Published:** 2019-04-11

**Authors:** Kristi Rüütel, Liis Lemsalu, Sirly Lätt, Jevgenia Epštein, on behalf of OptTEST by HiE

**Affiliations:** 1 National Institute for Health Development, Tallinn, Estonia; 2 Estonian Health Insurance Fund, Tallinn, Estonia; 3 Estonian Health Board, Tallinn, Estonia; 4 Optimising testing and linkage to care for HIV across Europe (http://www.opttest.eu/)

**Keywords:** Estonia, blood-borne infections, sexually transmitted infections, viral infections, HIV infection, human immunodeficiency virus – HIV, HIV testing, late diagnosis

## Abstract

**Background:**

Studies of missed opportunities for earlier diagnosis of HIV have shown that patients with undiagnosed HIV often present to healthcare settings numerous times before eventually receiving their diagnosis.

**Aim:**

The aim of the study was to assess missed opportunities for HIV testing among people newly diagnosed with HIV.

**Methods:**

In this observational retrospective study, we collected data from the Estonian Health Board on new HIV cases in people aged 16–49 years diagnosed in 2014–15 and from the Estonian Health Insurance Fund database for treatment invoices on their contacts with healthcare services in the 2 years preceding diagnosis. Diagnoses on treatment invoices were categorised as HIV indicator conditions using ICD-10 codes.

**Results:**

Of 538 newly diagnosed HIV cases (62.5%; 336 men), 82% had visited healthcare services at least once during the 2 years before HIV diagnosis; the mean number of visits was 9.1. Of these, 16% had been tested for HIV and 31% had at least one ICD-10 code for an HIV indicator condition on at least one of their treatment invoices. In 390 cases of HIV indicator conditions, only 5% were tested for HIV. Of all new HIV cases aged 20–49 years from high-incidence regions (defined as priority groups in national testing guidance), 18% had been tested.

**Conclusions:**

The HIV testing rate in the 2 years before an HIV diagnosis was very low, even in the presence of an HIV indicator condition. This emphasises the importance of implementing the Estonian HIV testing guidelines.

## Introduction

HIV testing is the gateway to HIV prevention, treatment, care and other support services [1]. Despite the widely acknowledged benefits of HIV testing, gaps remain in reducing the number of people living with HIV (PLHIV) who are unaware of their infection. Recent studies have suggested that the estimated proportion of PLHIV in the European Union (EU) and European Economic Area (EEA) who are undiagnosed is around 15% [2,3] and the average time between HIV infection and diagnosis is nearly 4 years [3]. EU countries are nearing the UNAIDS 90–90–90 target for the year 2020 and reducing the proportion of undiagnosed PLHIV remains the greatest barrier to achieving its target, suggesting that further efforts are needed to improve HIV testing rates [2].

Studies of missed opportunities for earlier diagnosis have shown that patients with undiagnosed HIV often present to healthcare settings numerous times before eventually receiving their diagnosis [4-6]. Many of them are not tested for HIV even if presenting with HIV indicator conditions (IC) [5,7]. HIV ICs are conditions which are AIDS-defining among PLHIV, conditions associated with an undiagnosed HIV prevalence of > 0.1%, or conditions where not identifying the presence of HIV infection may have significant adverse implications for the individual’s clinical management [8].

Estonia, located in north-eastern Europe, has a total population of ca 1.3 million [9] and one of the highest rates of newly diagnosed HIV cases in the EU (16.6 cases/100,000 population in 2017) [10]. Historically, the HIV epidemic has been concentrated among people who inject drugs, but heterosexual transmission and transmission among men who have sex with men (MSM) has increased in recent years [10]. In Estonia, HIV testing rates among the general population and people who inject drugs have been high. Approximately 12% of the total population is tested annually (119/1,000 population) [11]. Among people who inject drugs, up to 97% have tested for HIV during their lifetime, and up to 93% of those who are HIV-infected are aware of this [11]. Despite this, it is estimated that only 72% of PLHIV have been diagnosed [12]. Also, the proportion of concurrent AIDS has increased in recent years, from 2% in 2010 to 4% in 2017, and most likely underestimated [13]. Data on CD4 counts is available only at the point of linkage to HIV care, not at diagnosis. This data also indicates a high proportion of late diagnosis: as of 2013, 53% of PLHIV first present to care with CD4 counts of fewer than 350 cells/ml [14].

In Estonia, HIV testing has been available since 1987 and any doctor (whether a family practitioner or a specialist doctor) can recommend HIV testing based on clinical indications, risk assessment or the patient’s request [11]. During the period studied, HIV testing was free of charge only for people with national health insurance. People with no insurance could test free of charge at anonymous testing sites, which are usually affiliated to local hospitals [15]. Testing is available at drug-treatment and harm-reduction sites. In prisons, HIV testing is offered routinely during imprisonment with high testing rates when entering prison (97% in 2012) [16]. There are no special incentives for healthcare organisations to test. The latest national guidance for provider-initiated testing and counselling took effect in 2012. The main groups for whom HIV testing is recommended are people with HIV ICs, people who have injected drugs or had risky sex, pregnant women and prisoners. In the two regions with the highest HIV incidence (Harju County, including the capital, Tallinn, and Ida-Viru County in the north-east of the country), HIV testing is recommended for all people aged 16–49 years [17]. Previous research has identified large gaps in implementation of national testing guidance, including low testing rates in people with HIV ICs and in high-incidence regions [18,19].

To assess the situation in Estonia we aimed to study the pattern of healthcare visits and HIV testing 2 years before HIV diagnosis among people newly diagnosed with HIV in 2014–15.

## Methods

This study is an observational retrospective study implementing secondary analysis of the data from the Estonian Health Insurance Fund (EHIF) and Health Board (HB). The sample consists of patients aged ≥ 16 years who were diagnosed with HIV for the first time between 1 January 2014 and 31 December 2015.

### Data sources and definitions

From the HB communicable diseases information system, we collected the following data for the people newly diagnosed with HIV in 2014–15: (i) date of HIV diagnosis (confirmation date in national HIV reference laboratory); (ii) sex and age at the time of HIV diagnosis; (iii) place of residence at the time of HIV diagnosis (on county level); (iv) self-reported transmission mode (heterosexual/MSM/injecting drug use/other/unknown); (v) setting where HIV was diagnosed, categorised as one of the following: family practitioner (primary care); emergency medicine; infectious diseases clinics, including anonymous testing; gynaecology and obstetrics (including midwives); specialist doctors (all other specialist care); other (e.g. blood donors); prison; unknown.

Based on the unique national identification codes of the patients, HB data were linked with the data from EHIF’s database of treatment invoices. EHIF is the core purchaser of healthcare services in Estonia, covering healthcare costs for insured people. EHIF also manages services for people without health insurance (covered directly from the state budget). EHIF database does not include prison health services and anonymous testing. After healthcare service provision, the provider sends an invoice to EHIF, which includes doctor and patient information (e.g. age, sex, diagnosis based on the World Health Organization (WHO) International Statistical Classification of Diseases and Related Health Problems (ICD-10) [20]) and services provided (tests performed, etc.). Every test in the database has a specific code. An HIV test has had a separate code since 2012.

For people diagnosed with HIV in 2014, we extracted invoices from the period 2012–14 and for those diagnosed in 2015, for 2013–15. For every person, we included invoices which started ≤ 730 days (2 years) before HIV diagnosis (based on the start date of the invoice and the date of HIV diagnosis). We did not include invoices for which start/end date overlapped with HIV diagnosis date (assuming that the HIV test in these cases was the test leading to HIV diagnosis).

We did not include the invoices issued by the following specialties (as they either cannot order HIV tests or are not expected to do this): laboratory medicine, radiology, physiotherapy, ophthalmology, dentistry, medical genetics, psychology and nursing.

We used treatment invoices as a proxy for health care visits. The following data were extracted for every invoice from EHIF’s database: (i) invoice start and end dates which reflect the start and end for the health care services provided; (ii) insurance status (insured/not insured); (iii) specialty of the doctor issuing the invoice; (iv) diagnoses (ICD-10 codes); (v) HIV testing (yes/no; if yes, then the date of testing).

The specialty of the doctor issuing the invoice was categorised as following: family practitioner (primary care); emergency medicine; infectious diseases; gynaecology and obstetrics (incl. midwives); dermatovenerology; psychiatry; specialist doctors (all other specialist care); general practitioners (unspecialised doctors working mostly in hospitals but also in primary care).

The diagnoses on treatment invoices were categorised as HIV ICs as recommended by the HIV in Europe Initiative [8]: (i) cancers and neoplasms: C21, C34, C46, C53, C85–C89, C83, D15–D16, N87; (ii) infectious, fungal and parasitic diseases: A02.1, A15–A19, A31, A60, A81.2, A87.2–A87.9, B02, B15–B19, B25.9, B27, B55.0, A07.2, A07.3, B55, B57.2, B57.4, B58, B37, B39.0–B39.4, B45, B38.3–B38.9, B48.8; (iii) sexually transmitted infections (STI): A51–A64; (iv) pneumonia: J13, J15–J16, J18; (v) diseases of the blood and blood-forming organs: D72.8; (vi) diseases of the skin and subcutaneous tissue: L21, L40; (vii) diseases of the genitourinary system: N15.9; (viii) diseases of the nervous system: G90.0, G35, G56, G57, G59, G61.0; (ix) diseases of the digestive system: K13.3; (x) symptoms: R50 (fever of unknown origin), R63.4 (abnormal weight loss), R59.1 (generalised enlarged lymph nodes). We also included the group F11 (opioid-related disorders).

We refer to people in our final sample who had EHIF treatment invoices within 2 years before HIV diagnosis as the target group. We refer to people who were 20–49 years old at the time of HIV diagnosis (which means they were at least 18 during the 2 years before HIV diagnosis) and were diagnosed in Harju or Ida-Viru County as the priority groups (their universal testing is recommended in Estonian guidelines for HIV testing [17]).

The term ‘missed opportunity’ has no consensus definition [21]. For the purpose of our study we defined missed opportunity as a healthcare visit during which an HIV test was not performed to a patient with an HIV IC or which occurred in Harju or Ida-Viru County among those aged 16–49 years. EHIF data do not include information on risk behaviours so we were not able to follow these indications.

### Statistical analysis

The data were analysed using Microsoft Office Excel 2007 and Stata/IC 14.1. We used descriptive statistics to characterise participants and testing during healthcare visits. We compared categorical variables using the chi-squared or Fisher’s exact tests with a significance level of less than 0.05.

### Ethical statement

The study protocol was approved by the Tallinn Medical Research Ethics Committee (decision number 1408) and the Estonian Data Protection Agency (decision number 2.2-1/16/7).

## Results

### Characteristics of the sample

A total of 561 new HIV cases were reported to the HB in 2014–15. We excluded 23 cases for the following reasons: no unique national identification code, younger than 16 years of age, previously diagnosed with HIV (Figure).

**Figure:**
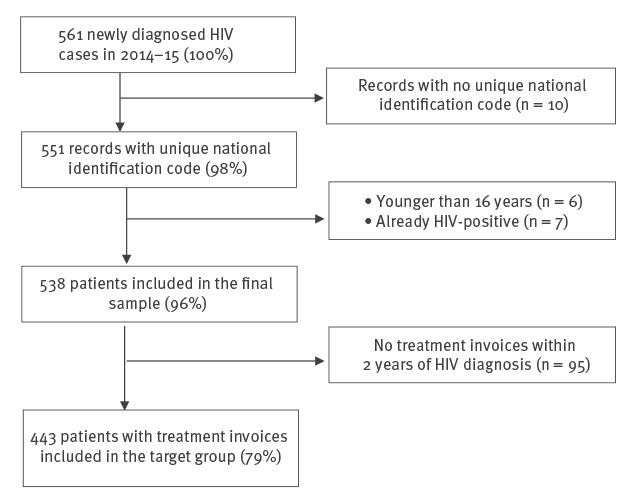
Construction of the sampling frame, study to assess missed opportunities for HIV testing among newly diagnosed HIV cases, Estonia, 2014–2015

The final sample consisted of 538 adults with an average age of 36.3 years at the time of HIV diagnosis: 63% were men, 42% were diagnosed with HIV in Ida-Viru County and 50% in Harju County. Of the 538, 56% had been infected through heterosexual contact, 23% through injecting drug use, and 4% were MSM (Table 1).

**Table 1:** Characteristics of HIV-infected patients, by year of HIV diagnosis, Estonia, 2014–2015 (n = 538)

Patient characteristics	Overall (n = 538)		Year of HIV-infection diagnosis				p value
			2014 (n = 275)		2015 (n = 263)		
	n	%	n	%	n	%	
**Age at HIV infection diagnosis (years)**							
Mean (median, SD, range)	36.3 (34; 10.7; 16–70)		36.6 (34; 10.6; 19–70)		35.9 (34; 10.8; 16–66)		0.5
**Age group (years)**							
< 30	167	31.0	81	29.5	86	32.7	0.5
30–39	197	36.6	99	36.0	98	37.3	
> 39	174	32.4	95	34.5	79	30.0	
**Sex**							
Male	336	62.5	174	63.3	162	61.6	0.7
Female	202	37.5	101	36.7	101	38.4	
**Estonian region where HIV was diagnosed**							
Ida-Viru county	228	42.4	119	43.3	109	41.4	0.8
Harju county	267	49.6	133	48.4	134	51.0	
Rest of Estonia	43	8.0	23	8.3	20	7.6	
**Setting of HIV diagnosis**							
Family practitioner	41	7.6	20	7.3	21	8.0	< 0.001
Emergency medicine	21	3.9	9	3.3	12	4.5	
Infectious diseases clinics, including anonymous testing	177	32.9	85	48.0	92	52.0	
Gynaecology and obstetrics	50	9.3	22	8.0	28	10.6	
Specialist doctors	132	24.5	69	25.1	63	24.0	
Other	10	1.9	3	1.1	7	2.7	
Prison	59	11.0	31	11.3	28	10.7	
Unknown	48	8.9	36	13.1	12	4.5	
**HIV transmission mode**							
Heterosexual	302	56.1	160	58.2	142	54.0	0.005
MSM	21	3.9	3	1.1	18	6.8	
Injecting drug use	122	22.7	67	24.4	55	20.9	
Unknown	93	17.3	45	16.3	48	18.3	

MSM: men who have sex with men; SD: standard deviation.

### Contact with the healthcare system during the 2 years before HIV diagnosis

The total number of invoices was 4,046. The average number of invoices per person in the final sample (n = 538) was 7.5 (median: 6; range: 0–68). Almost three quarters of those in the final sample (71%) had visited a family practitioner at least once (n = 383).

Of the final sample, 443 (82%) had EHIF treatment invoices within 2 years before HIV diagnosis (the target group), with an average of 9.1 invoices per person (median: 7; range: 1–68). Of the target group, 331 (75%) had invoices from family practitioners and specialist doctors, 56 (13%) from specialist doctors only, and 52 (12%) from family practitioners only. Four people (1%) had only emergency medicine invoices.

Women were more likely to have visited healthcare services than men, people younger than 39 years of age more so than older ones. People diagnosed with HIV in prisons had had fewer healthcare encounters in civil system (Table 2). Of all women (n = 202), 55% had visited a gynaecologist or a midwife at least once within 2 years before their HIV diagnosis (18% of all healthcare visits among women involved in the study).

**Table 2:** Characteristics of HIV-infected patients, by treatment invoices within 2 years of HIV diagnosis, Estonia, 2014–2015 (n = 538)

Patient characteristics	Treatment invoices				p value
	No (n = 95)		Yes^a^ (n = 443)		
	n	%	n	%	
**Sex**					
Male	73	21.7	263	78.3	0.001
Female	22	10.9	180	89.1	
**Age group (years)**					
< 30	14	8.4	153	91.6	0.001
30–39	44	22.3	153	77.7	
> 39	37	21.3	137	78.7	
**Estonian region where HIV was diagnosed**					
Ida-Viru county	38	16.7	190	83.3	0.8
Harju county	50	18.7	217	81.3	
Rest of Estonia	7	16.3	36	83.7	
**Setting of HIV diagnosis**					
Family practitioner	3	7.3	38	92.7	0.003
Emergency medicine	4	19.1	17	80.9	
Infectious diseases clinics, including anonymous testing	38	21.5	139	78.5	
Gynaecology and obstetrics	4	8.0	46	92.0	
Specialist doctors	16	12.1	116	87.9	
Other	1	10.0	9	90.0	
Prison	20	33.9	39	66.1	
Unknown	9	18.8	39	81.2	
**HIV transmission mode**					
Heterosexual	43	14.2	259	85.8	0.06
MSM	3	14.3	18	85.7	
Injecting drug use	25	20.5	97	79.5	
Unknown	24	25.8	69	74.2	

MSM: men who have sex with men.

^a^ The target group.

### HIV testing during the 2 years before HIV diagnosis

Out of the people in the target group, 72 (16%; 13% of the final sample) had been tested for HIV during the 2 years preceding HIV diagnosis. Women had been tested more often than men (24% vs 11%) and younger age groups more than older age groups (Table 3). Of the 72 people in the target group, 46 had been tested once, and 26 had been tested twice or more. The mean time from the last HIV test to HIV diagnosis was 408 days (median: 411 days; range: 21–721 days). Sixteen people had their previous HIV test within 6 months of HIV diagnosis and 32 within 1 year of HIV diagnosis.

**Table 3:** Characteristics of HIV-infected patients with treatment invoices within 2 years of HIV diagnosis by HIV testing, Estonia, 2014–2015 (n = 443)

Patient characteristics	Not tested (n = 371)		Tested (n = 72)		p value
	n	%	n	%	
**Sex**					
Male	234	89.0	29	11.0	< 0.001
Female	137	76.1	43	23.9	
**Age group (years)**					
< 30	121	79.1	32	20.9	0.05
30–39	127	83.0	26	17.0	
> 39	123	89.8	14	10.2	
**Estonian region where HIV was diagnosed**					
Ida-Viru county	162	85.3	28	14.7	0.7
Harju county	178	82.0	39	18.0	
Rest of Estonia	31	86.1	5	13.9	
**HIV transmission mode**					
Heterosexual	218	84.2	41	15.8	0.9
MSM	14	77.8	4	22.2	
Injecting drug use	82	84.5	15	15.5	
Unknown	57	82.6	12	17.4	
**Year of HIV diagnosis**					
2014	191	83.0	39	17.0	0.7
2015	180	84.5	33	15.5	

MSM: men who have sex with men.

The specialties with the largest number of visitors tested (data not shown in tables) were infectious diseases (12 people out of 25 tested at least once; 48% of those who had visited this specialty), and gynaecology and obstetrics (31 people out of 112 tested at least once; 28% of those who had visited this specialty). The lowest percentage of tested patients was in primary care (13 people out of 383; 3% of those who had visited this specialty).

### HIV testing in patients with HIV indicator conditions

Of the target group, 137 patients (31%) had at least one ICD-10 code for HIV IC on at least one of their treatment invoices. People who had an IC on at least one invoice did not differ from those who had none by age, sex and HIV transmission group (p > 0.5). Fewer people had ICs in Ida-Viru (46/190; 24%; p = 0.04) compared with Harju County (78/217; 35%) and the rest of Estonia (12/36; 33%).

Of the 137, 12 people (9%) had been HIV tested at least once when they had an IC. Some people had several treatment invoices (proxy for healthcare visit) and different ICs. Altogether, in 390 cases the treatment invoice included ICD-10 code(s) for HIV ICs (Table 4). Of these, 5% (n = 20) included an HIV test. The largest number of invoices with ICs was from family practitioners (n = 202); out of these only one included an HIV test (Table 4). Most common ICs were certain infectious diseases and opioid use (Table 5).

**Table 4:** Treatment invoices (n = 4,046) according to HIV indicator conditions and HIV testing by setting, patients diagnosed with HIV, Estonia, 2014–2015 (n = 434)

Setting	Treatment invoices with HIV indicator conditions			Treatment invoices with no HIV indicator conditions			p value
	Total	With HIV test		Total	With HIV test		
	n	n	%	n	n	%	
Family practitioners	202	1	0.5	1,822	13	0.7	0.7
Emergency medicine	12	1	8.3	92	7	7.6	0.9
Infectious diseases	18	10	55.6	20	7	35.0	0.2
Dermatovenerology	32	1	3.1	110	3	2.7	0.9
Psychiatry	29	2	6.9	213	1	0.5	0.003
Gynaecology and obstetrics	39	2	5.1	353	41	11.6	0.2
Other specialist doctors	46	1	2.2	791	10	1.3	0.6
General practitioners^a^	12	2	16.7	255	11	4.3	0.05
Total	390	20	5.1	3,656	93	2.5	0.003

^a^ The specialty of the doctor issuing the invoice was categorised differently to the settings where HIV was diagnosed (see methodology).

**Table 5:** Treatment invoices with HIV indicator conditions among people diagnosed with HIV^a^ within 2 years of HIV diagnosis, Estonia, 2014–2015 (n = 434)

HIV indicator condition group	All invoices with ICs		Invoices with ICs with HIV tests	
	n	Proportion of all invoices^a^ %	n	Proportion of all invoices with respective ICs %
Certain infectious, fungal and parasitic diseases	165	4.1	14	8.5
Pneumonia	24	0.6	0	0
HIV-related symptoms	16	0.4	1	6.3
Sexually transmitted infections	21	0.5	2	9.5
Cancers and neoplasms	2	0.05	1	50.0
Diseases of the skin	46	1.1	1	2.2
Diseases of the nervous system	15	0.4	0	0
Mental and behavioural disorders due to use of opioids	110	2.7	1	0.9
**Total**	**390**^b^	**9.6**	**20**	**5.1**

IC: indicator condition.

^a^ Total number of invoices n = 4,046.

^b^ 381 invoices had one indicator condition (IC) and nine invoices had two IC ICD-10 codes.

### HIV testing in patients belonging to the priority group

Of the target group, 356 (80%) were 20–49 years old at the time of the HIV diagnosis and were diagnosed in Harju or Ida-Viru County. These factors placed them in the priority groups set in the Estonian guidelines for HIV testing. Their average number of treatment invoices was 8.9 (median: 7; range: 1–68). Of these 356, 304 (85%) had visited family practitioner at least once. The total number of family practitioner visits was 1,577 and during these visits, HIV tests were performed only 13 times (0.8%).

Of those in the priority groups, 65 (18%) had been tested for HIV at least once. They were more likely to have been tested than those over 49 years of age or from other regions of Estonia (8%; p = 0.02). They had an HIV IC on their treatment invoice as often as others (31% vs 31%; p = 0.9). Those with an HIV IC were tested more often than people with ICs who did not belong to the priority group (29% vs 13%; p = 0.1).

## Discussion

Increased HIV-related morbidity and mortality, poorer response to treatment and increased healthcare costs are the consequences of late HIV diagnosis. Moreover, delayed diagnosis is one of the most important determinants of increased rates of HIV transmission [22]. Our data confirms that many HIV-infected patients in Estonia make numerous visits to healthcare services before being tested. In our study, 82% of the new HIV cases had visited healthcare services at least once in the 2 years before HIV diagnosis and the mean number of their visits was 9.1. We found evidence of HIV testing for 16% of them. Only 13% of all new HIV cases had been tested in a healthcare setting. Thus, missed opportunities for testing in healthcare contribute to the late diagnosis of HIV in Estonia.

Of all new HIV cases, 72% had visited a family practitioner at least once but only 3% of them had been tested for HIV at least once. Also, less than 1% of the visits from people belonging to HIV testing priority groups resulted in HIV testing at family medicine practices. This is an extremely low level of testing. Considering that primary care is the most-visited setting, testing should be scaled up by ensuring proper funding for family practitioners to test everyone in need, including people with no health insurance, and possibly also introducing incentives for healthcare personnel to encourage testing.

In our study, 95% of cases with ICs were not tested for HIV. Even in medical professions like dermatovenerology, only 3% of patients with ICs were tested for HIV. This has also been seen in other countries. In the United States, in New York City, many patients being evaluated for gonorrhoea and chlamydia failed to receive HIV testing, especially in emergency and inpatient settings. The testing rates were, however, much higher than in our study (in 2015, 70% of men and 51% of women received same-day HIV testing) [23]. In the Netherlands, a study among PLHIV revealed that in one third of the STI-related consultations for persons from high-risk groups, no HIV test was performed in primary care [7].

Also, of all new cases aged 20–49 years from high incidence regions, 82% had not been tested for HIV despite being in the priority group. HIV testing among them was mostly related to women being tested in gynaecology and obstetrics (opt-out testing twice during pregnancy is recommended by law). This low level of testing suggests that adherence to the Estonian HIV testing guidance is very low in recommended populations, conditions and settings.

Two thirds of our patients had no record of HIV ICs in the 2 years before HIV diagnosis. This has also been seen in other settings suggesting that symptomatology is not a reliable criterion to prompt HIV testing [24] and that routine testing is needed before patients present to care with symptoms suggestive of HIV [25].

Of the newly diagnosed HIV cases, 18% had no evidence of contact with healthcare services before HIV diagnosis (the proportion was higher among men and people aged ≥ 30 years, and those diagnosed with HIV in prisons). This does not necessarily mean that they had not been tested in the previous 2 years. In Estonia, it is also possible to test anonymously, which is not reflected in the EHIF’s database. People diagnosed in prisons had fewer healthcare visits. One reason is incarceration itself (they were in prison for at least part of the 2 years before HIV diagnosis), another reason may be that incarcerated people are likely to belong to more vulnerable population groups with fewer healthcare encounters in general. In Estonian prisons, HIV testing is offered routinely upon and during imprisonment [16], resulting in their diagnosis in the prison setting.

Different definitions of ‘missed opportunity’ have been used by different authors, usually reflecting local HIV testing guidance. Also, the timespan under evaluation has been different, most often being 1 [4,6,26,27], 3 [5,24], or 5 years before HIV diagnosis [21,25]. As a result, the proportions of missed opportunities for HIV testing in the medical literature range from 15% to 80%. We chose a 2-year period, because HIV testing could be tracked in EHIF database since 2012. Unlike some other studies [21,25], we were not able to track the need for testing based on risk behaviours. For example, a Swiss study revealed that 59% of missed opportunities were in people at epidemiological risk of acquiring HIV (belonging to or having a sexual partner from a high-risk group) [21]. Thus, the missed opportunities in our study may be underestimated.

### Limitations

Our data has several limitations. The EHIF database’s primary function is to track healthcare costs and not necessarily to provide information on the quality of healthcare. ICs as well as HIV tests may have been miscoded on treatment invoices. HIV tests can be marked on the invoice not only as ‘HIV test’ but also as ‘detection of infection marker using immunological methods’. The price for these two tests has always been the same, thus for tracking of healthcare costs it has not been important to distinguish which one is marked on the invoice. This misclassification would tend to increase rather than decrease the proportion tested.

We have no information about the HIV test offer and thus the proportion refusing testing. In Estonia, the only group tested mandatorily are blood and organ donors [11]. There is no information on refusal rates in any setting.

We have no data on private healthcare services (paid by patients themselves or by other insurance companies) and HIV testing therein. Considering that 94% of the population has national health insurance (87% of 20–49-year-olds), the proportion of these services is small. We also have no data on testing of these patients in prisons as well as anonymous testing sites. A relatively large proportion of new HIV cases diagnosed in the past decade have been found in both these settings [11,15].

### Conclusions

Missed opportunities for HIV testing among newly diagnosed HIV cases were numerous in Estonia. Therefore, it is critical to follow Estonian HIV testing guidelines, paying special attention to testing men aged 16–49 years and living in high-incidence areas. Primary care had been visited by almost three quarters of patients but testing rates there were extremely low; this is one of the most important settings in which to promote testing. While almost one fifth of our sample had no healthcare encounters before HIV diagnosis, offering testing in alternative settings (community-based organisations, etc.) is also needed. Considering the limitations, we recommend a clinical audit based on patient records in order to evaluate the implementation of the HIV testing guidance.
